# Supplementary material for: Misexpression of inactive genes in whole blood is associated with nearby rare structural variants
Source: Am J Hum Genet. 2024 Jul 24;111(8):1524–43. doi: 10.1016/j.ajhg.2024.06.017 (PMC11339615; doi:10.1016/j.ajhg.2024.06.017)
Supplement: Document S1. Figures S1–S15 [file mmc1.pdf]

The American Journal of Human Genetics, Volume *111*

Supplemental information

Misexpression of inactive genes in whole blood is associated with nearby rare structural variants

Thomas Vanderstichele, Katie L. Burnham, Niek de Klein, Manuel Tardaguila, Brittany Howell, Klaudia Walter, Kousik Kundu, Jonas Koeppel, Wanseon Lee, Alex Tokolyi, Elodie Persyn, Artika P. Nath, Jonathan Marten, Slavé Petrovski, David J. Roberts, Emanuele Di Angelantonio, John Danesh, Alix Berton, Adam Platt, Adam S. Butterworth, Nicole Soranzo, Leopold Parts, Michael Inouye, Dirk S. Paul, and Emma E. Davenport

Supplemental Figures

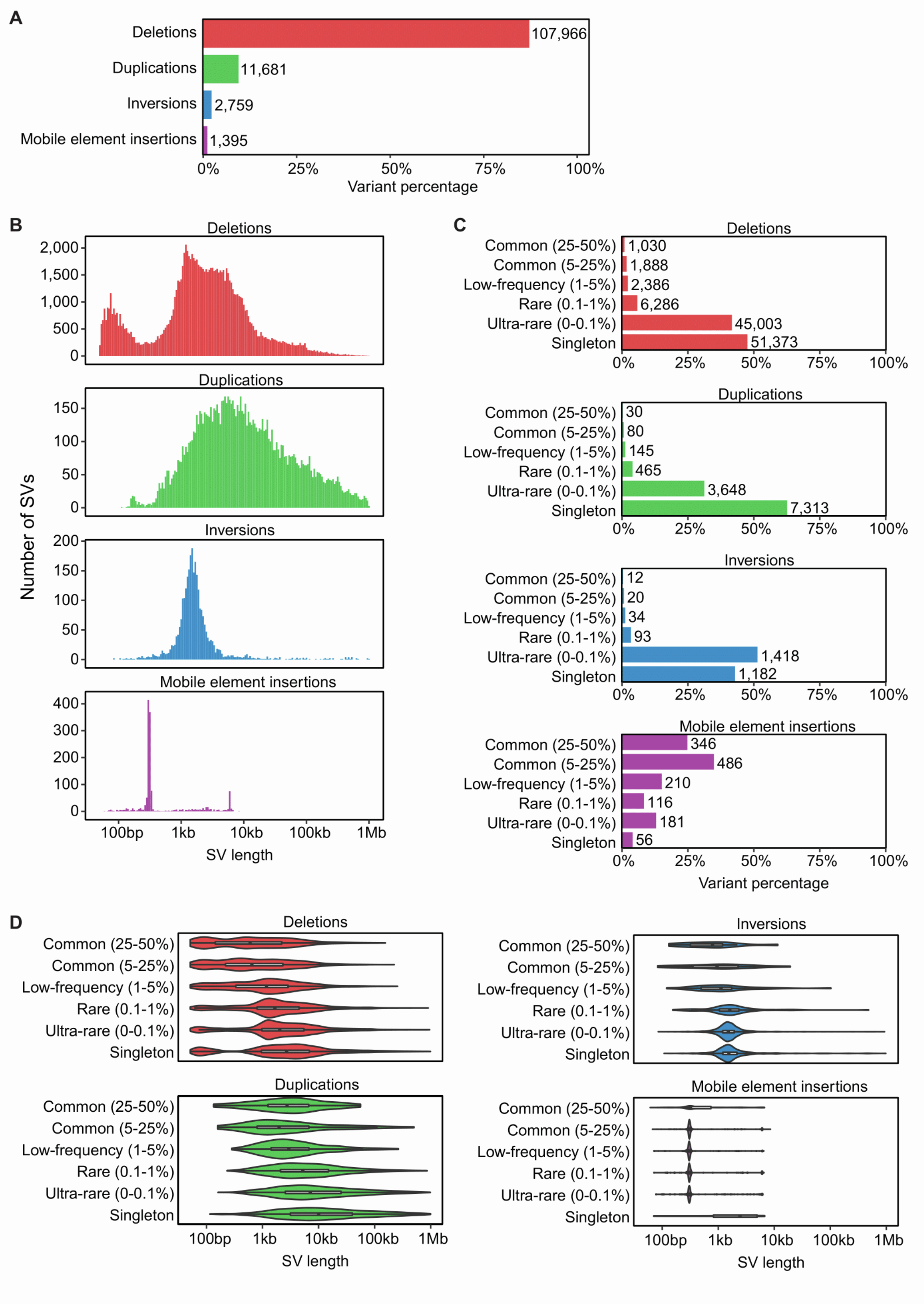

Figure S1. Overview of the SV callset.
A.) The percentage of different SV classes in the callset. Text labels indicate the total number of SV calls for each SV class. **B.)** Histogram showing the number of SVs across different length bins (log-transformed) stratified by SV class. **C.)** The percentage of SVs within each allele frequency bin by SV class. Text labels indicate the number of SV calls within each allele frequency bin. **D.)** The length distribution of SVs within each allele frequency bin by SV class. The lower, middle and upper hinges of the box plots correspond to the 25th percentile, median and 75th percentile, respectively.

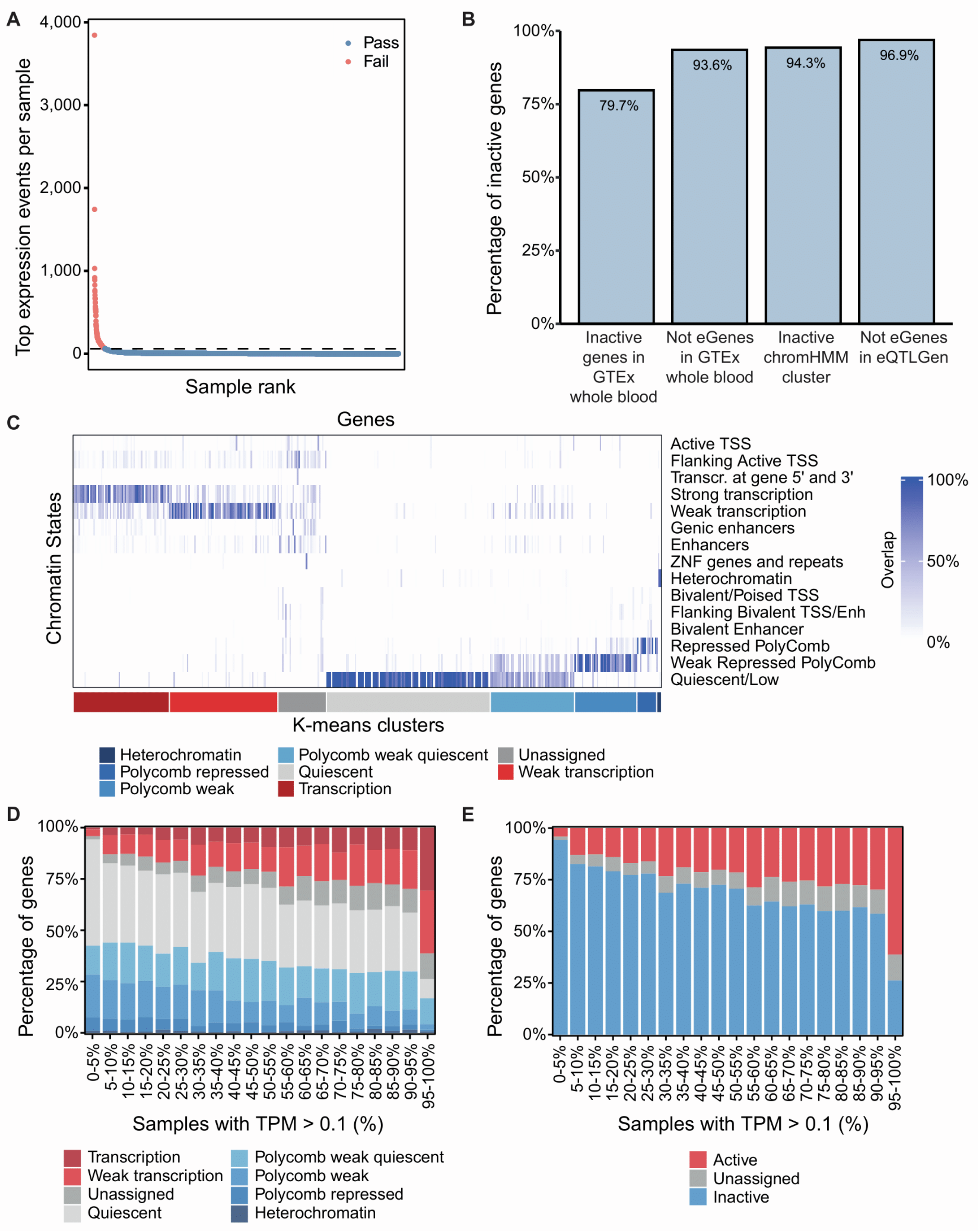

Figure S2. Removal of global expression outliers and inactive gene set validation.
A.) Number of top expression events (y-axis) ranked across all samples (x-axis). The dashed line indicates the threshold for removing aberrant samples. Failed samples (red) had a greater number of top expression events than this threshold while samples passing (blue) had a lower number. **B.)** Different inactive gene validation approaches showing the percentage of inactive genes identified in INTERVAL (y-axis) within different gene sets (x-axis). **C.)** Heatmap showing the percentage overlap of 60,603 genes (x-axis) over 15 chromHMM states from PBMC data. Genes are clustered into 8 k-means clusters and each cluster is labeled according to the types of overlapping states. **D.)** Percentage of genes in each k-means cluster stratified by gene expression activity. For each gene, expression activity

is quantified as the percentage of samples where the gene has a TPM > 0.1 (x-axis). **E.)** Percentage of genes labeled as active, inactive or unassigned from chromHMM k-means clusters stratified by gene expression activity. For each gene, activity is quantified as the percentage of samples where the gene has a TPM > 0.1 (x-axis). Inactive genes are defined as having a TPM > 0.1 in less than 5% of samples.

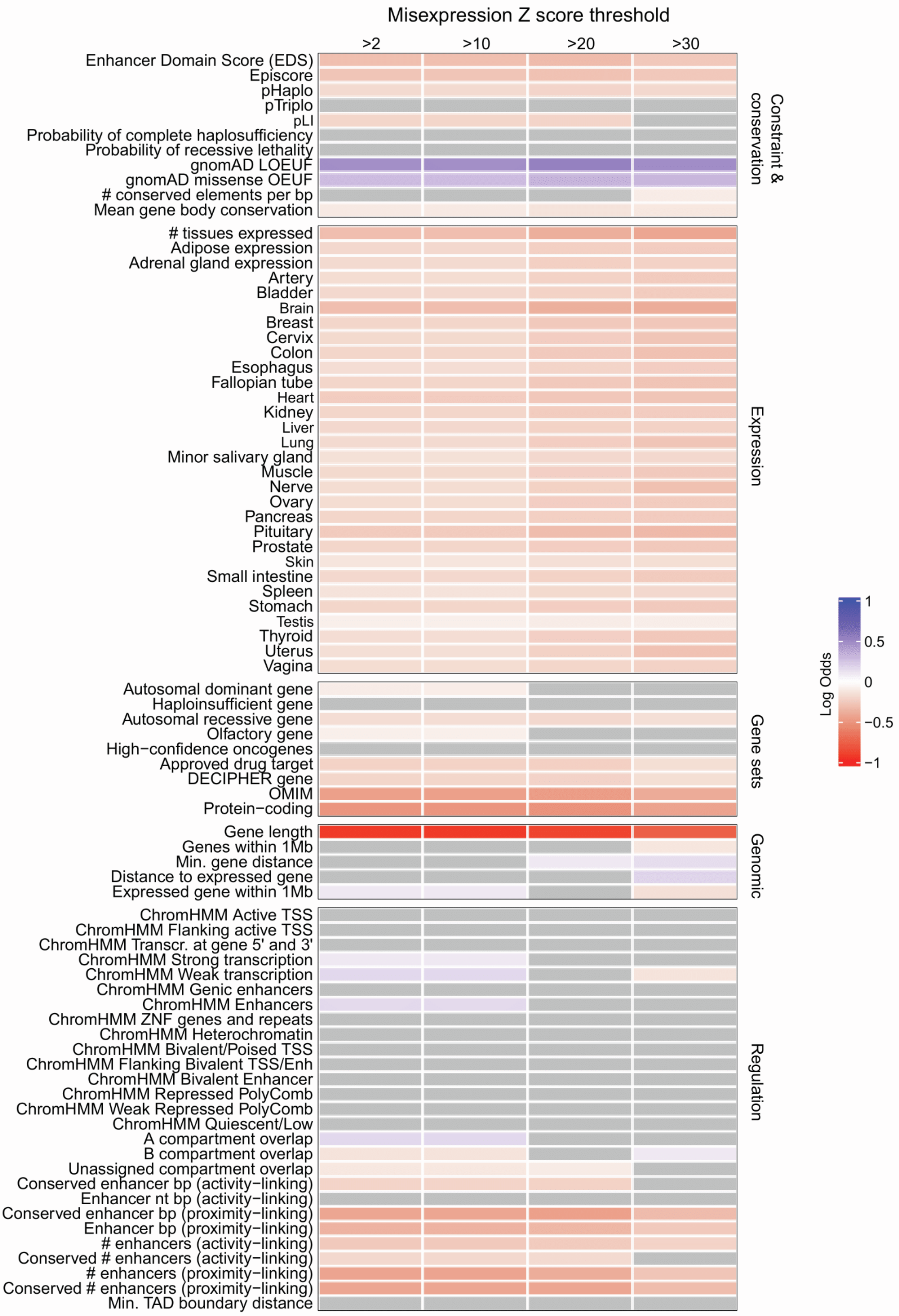

Figure S3. Different properties of misexpressed and non-misexpressed genes across misexpression Z score thresholds.

Enrichment of all 82 gene-level features within genes that are misexpressed versus non-misexpressed genes across different misexpression Z score thresholds. Features are grouped into different categories. Tiles shaded in gray do not pass a Bonferroni-adjusted p-value threshold ($p < 0.05$).

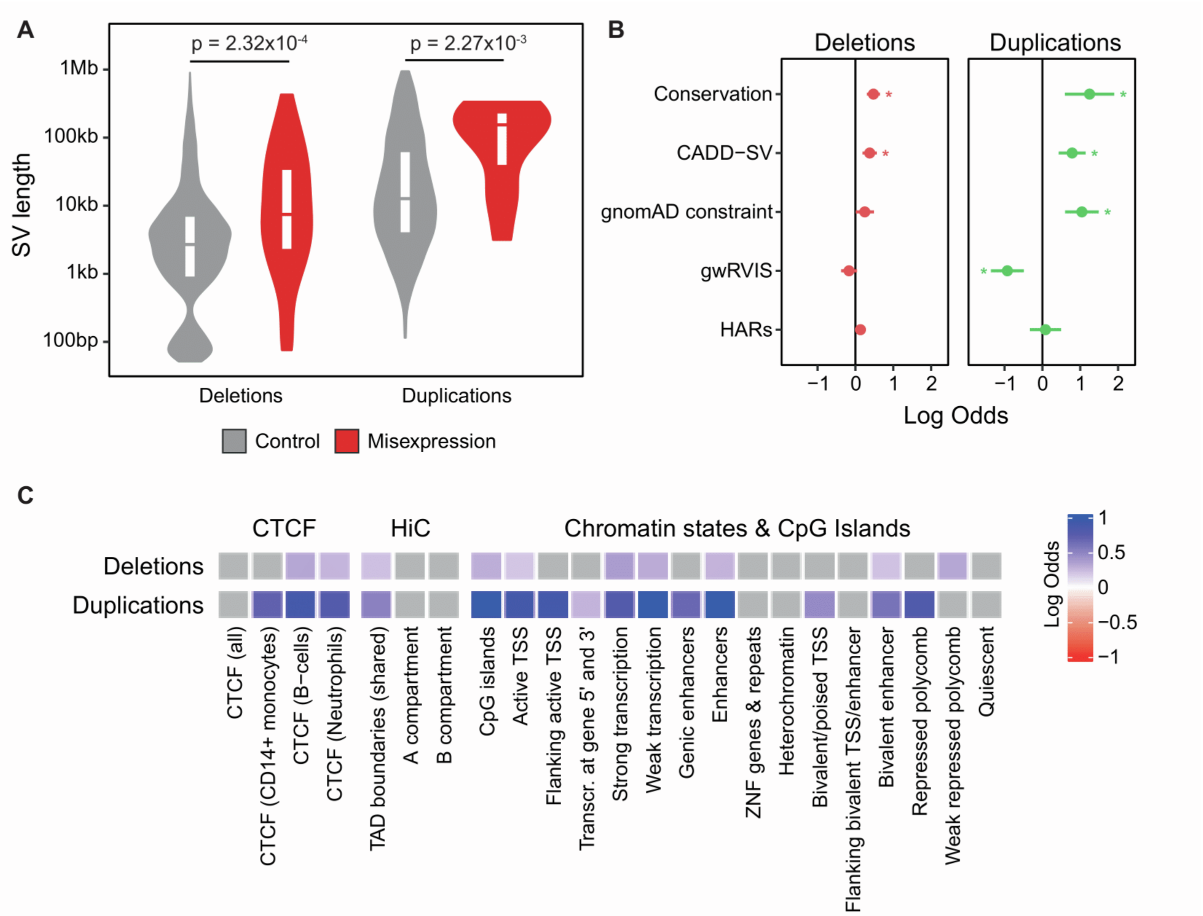

Figure S4. Properties of misexpression-associated rare SVs.

A.) SV length distributions of misexpression-associated and control duplications and deletions restricted to singletons only. The lower, middle and upper hinges of the box plots correspond to the 25th percentile, median and 75th percentile, respectively. P values were calculated using a one-sided Mann-Whitney test comparing the lengths of control and misexpression-associated SVs **B.)** Enrichment (x-axis) without adjusting for SV length of misexpression-associated deletions (left panel, red) and duplications (right panel, green) compared to controls for genomic scores (y-axis) including evolutionary conservation (phyloP), predicted deleteriousness (CADD-SV), constraint (gnomAD Z score constraint and gwRVIS), and HARs. Enrichments were calculated as the log odds ratio with lines indicating 95% confidence intervals for the fitted parameters using the standard normal distribution. Asterisks indicate significant enrichment after Bonferroni correction. **C.)** Enrichment without adjusting for SV length of misexpression-associated deletions and duplications compared to controls for regulatory features including CTCF candidate cis-regulatory elements from ENCODE, TAD boundaries shared across multiple cell-lines, A and B compartments, chromatin states from the Roadmap Epigenomics Project and CpG islands from the UCSC genome browser. Enrichments were calculated as the log odds ratio and tiles shaded in gray do not pass Bonferroni correction.

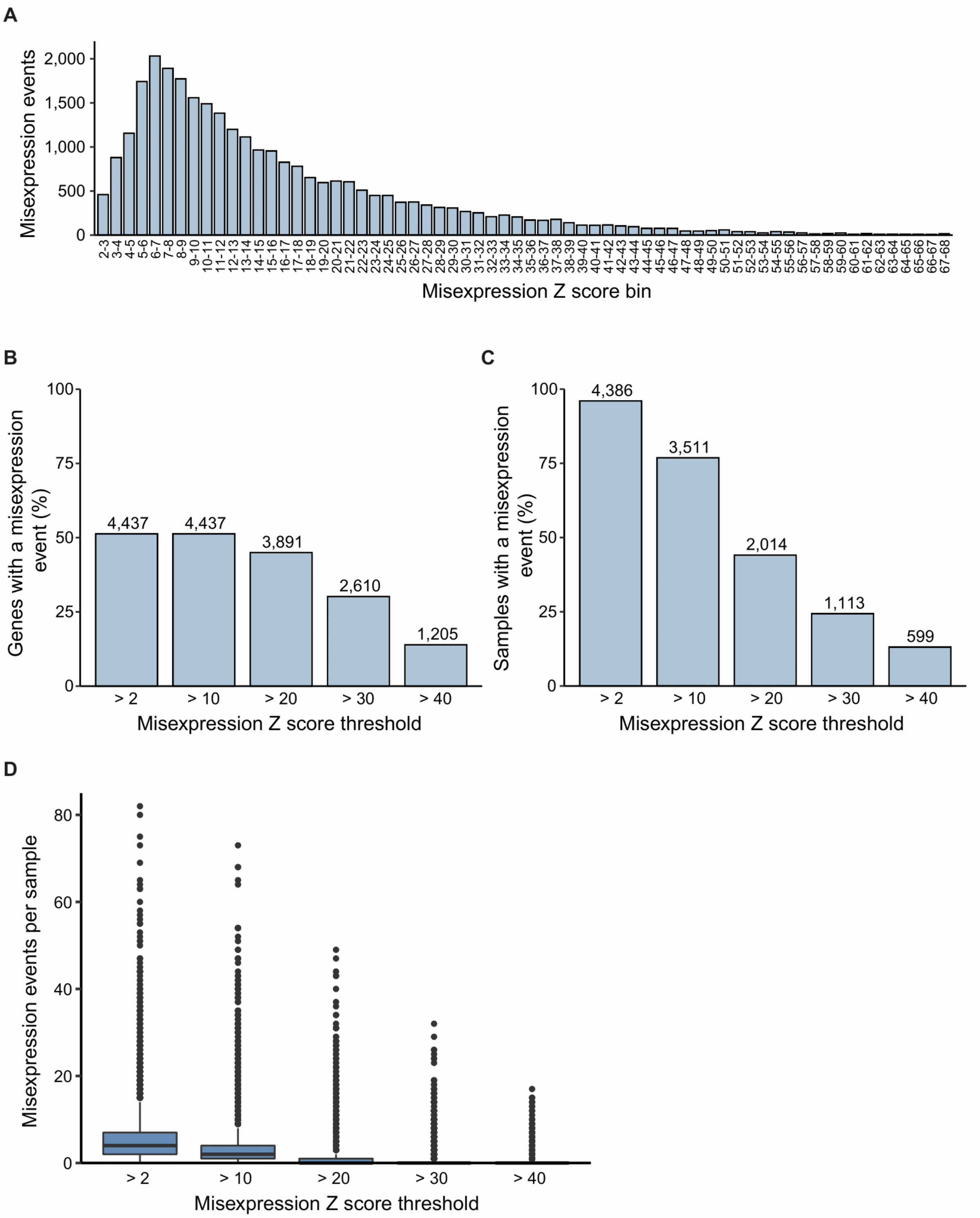

Figure S5. Misexpression metrics across genes and samples.

A.) Number of misexpression events across different misexpression Z score threshold bins. **B.)** Percentage of 8,650 inactive genes that have at least one misexpression event across different misexpression Z score thresholds. Text labels indicate the total number of genes with at least one misexpression event. **C.)** Percentage of 4,568 samples that have at least one misexpression event across different misexpression Z score thresholds. Text labels indicate the total number of samples with at least one misexpression event. **D.)** Number of misexpression events per sample across different misexpression Z score thresholds.

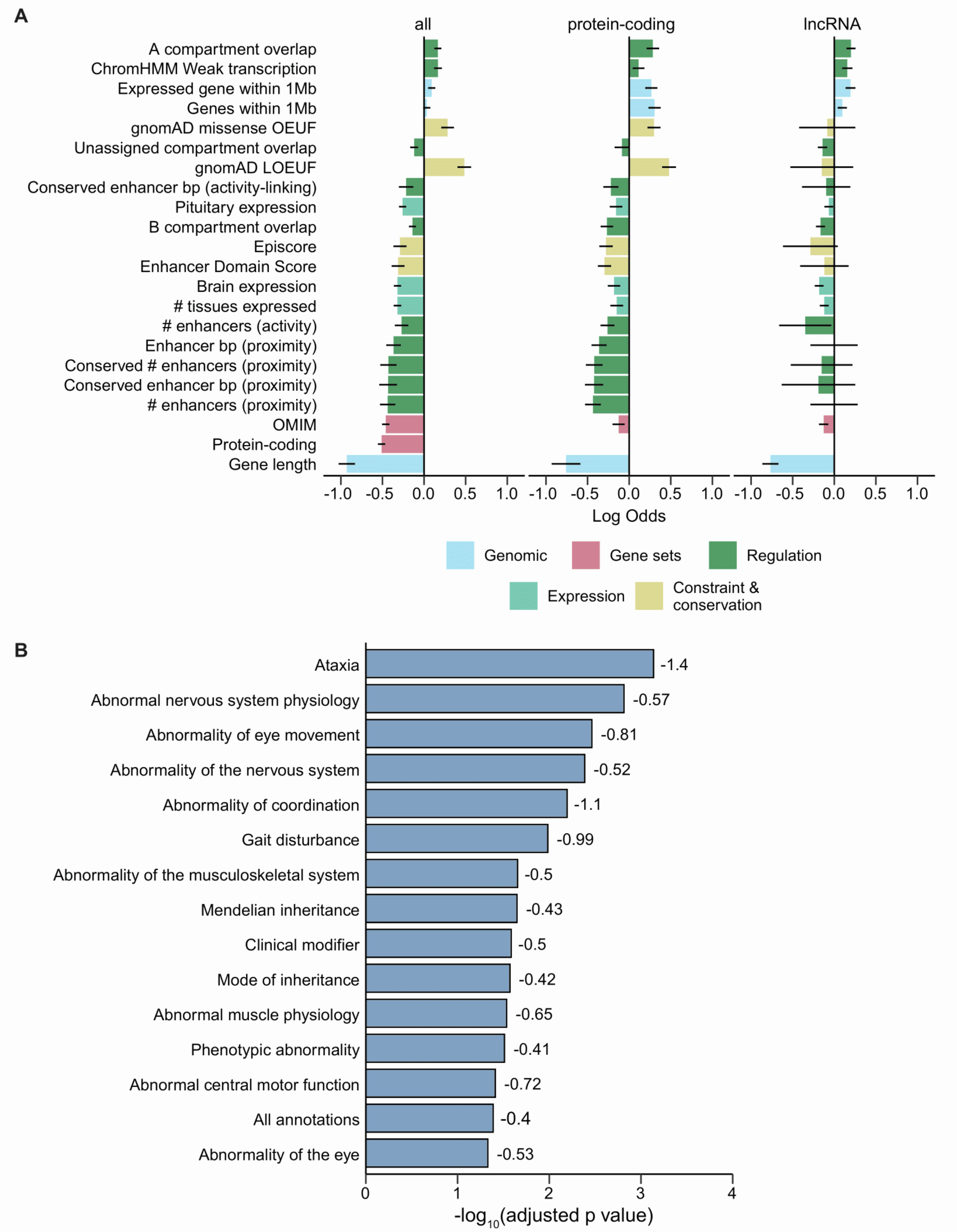

Figure S6. Properties of misexpressed genes.

A.) Enrichment of gene-level features within genes that are misexpressed (Z score > 2 and TPM > 0.5) versus non-misexpressed genes split across all genes, protein-coding genes and lncRNA genes. The union of the top 15 features with the highest absolute log odds passing Bonferroni correction within each gene set are shown. Lines indicate 95% confidence intervals for the fitted parameters using the standard normal distribution. **B.)** Human Phenotype Ontology (HPO) terms, by $-\log_{10}$(adjusted p-value) on the x-axis, underrepresented within 1,070 misexpressed protein-coding genes using 3,092 inactive protein-coding genes as the custom background. Only significant terms are shown. The log-odds ratio

for each term is shown adjacent to each bar. Negative log odds ratio indicates under enrichment in misexpressed genes.

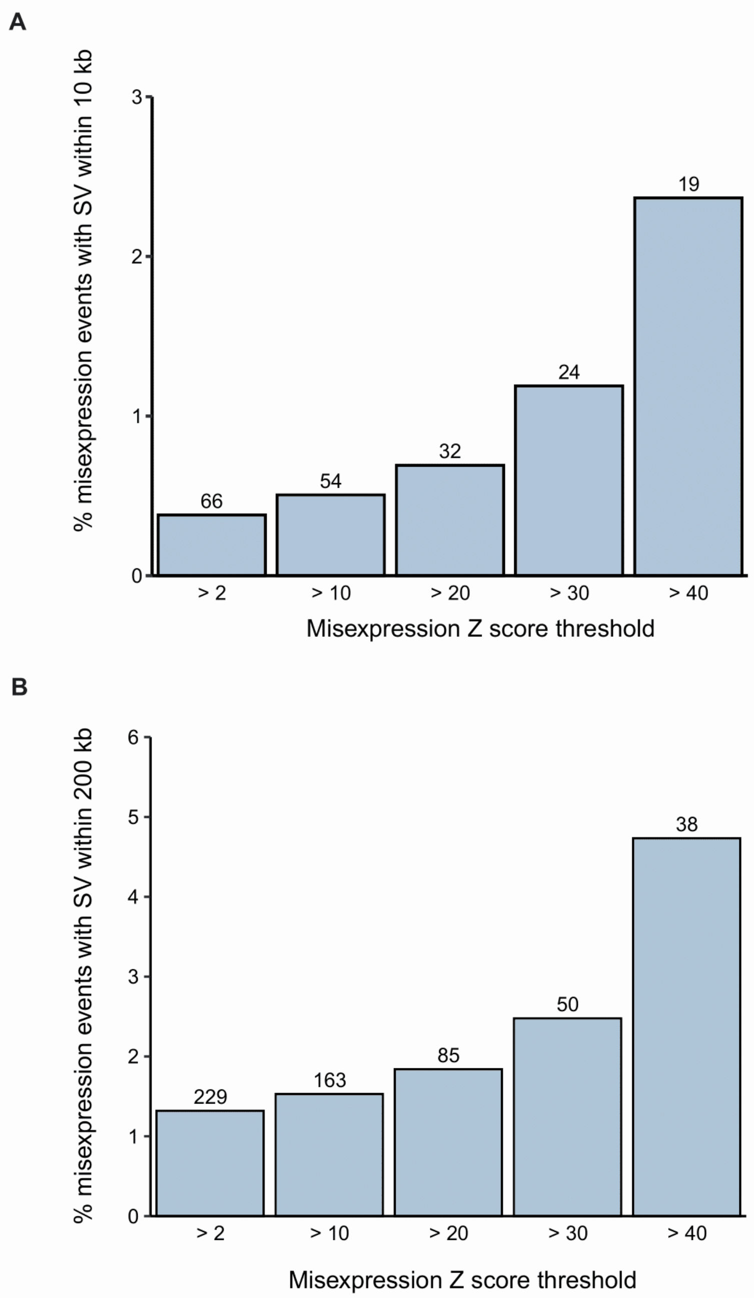

Figure S7. Percentage of misexpression events with a rare SV within 10 kb and 200 kb. Percentage of misexpression events (y-axis) with a rare SV within **A.)** 10 kb and **B.)** 200 kb at different misexpression Z score thresholds (x-axis). Text labels indicate the total number of misexpression events with an SV.

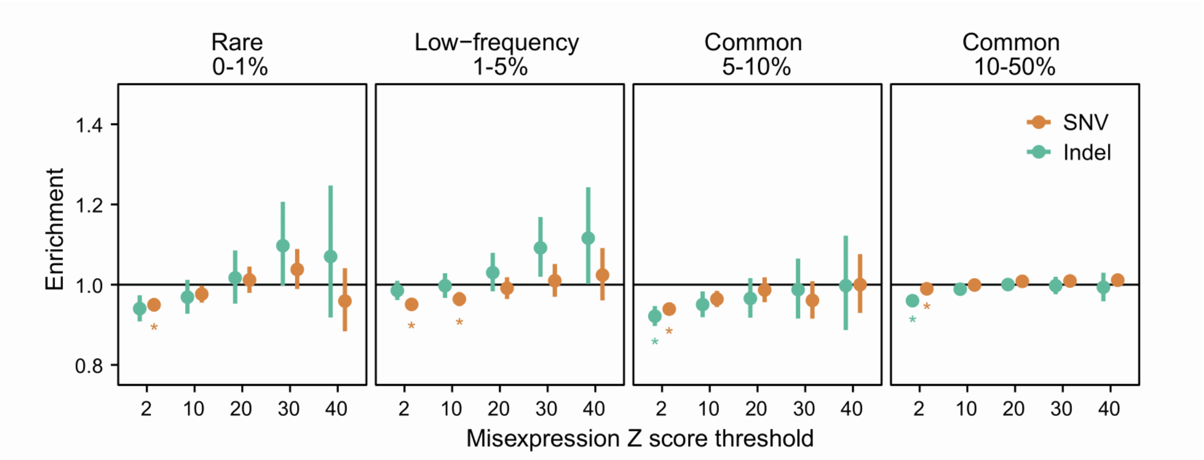

Figure S8. Enrichment of SNVs and indels within the gene body and flanking sequence of genes involved in misexpression events across different misexpression Z score thresholds and MAF cutoffs.

A flanking sequence of ±10 kb was used for SNVs and indels. Enrichments were calculated as the relative risk of having a nearby variant type given the misexpression status. Bars represent 95% Wald confidence intervals of the relative risk estimates. The line at enrichment = 1 indicates no enrichment; asterisks positioned either side of the line indicate significant enrichment or underenrichment after Bonferroni correction.

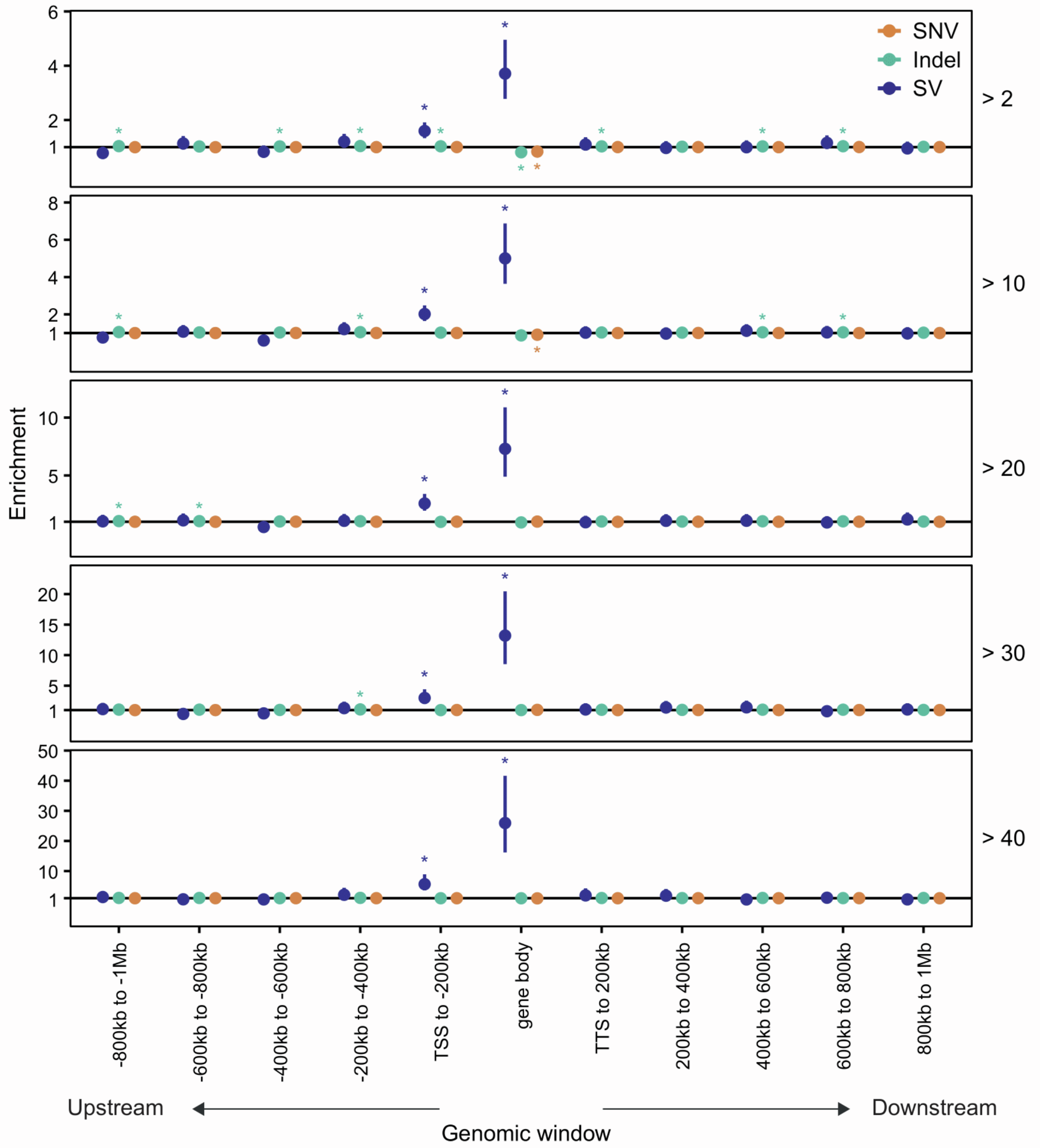

Figure S9. Enrichment of rare SNVs, indels and SVs across genomic windows and misexpression Z score thresholds.

Enrichment of rare (MAF < 1%) SNVs (orange), indels (green) and SVs (blue) within 200 kb genomic windows and the body of the misexpressed gene across different misexpression Z score thresholds. Enrichments were calculated as the relative risk of having a nearby rare variant type given the misexpression status. The line at enrichment = 1 indicates no enrichment; Asterisks positioned either side of the line indicate significant enrichment or underenrichment after Bonferroni correction. Bars represent 95% Wald confidence intervals of the relative risk estimates.

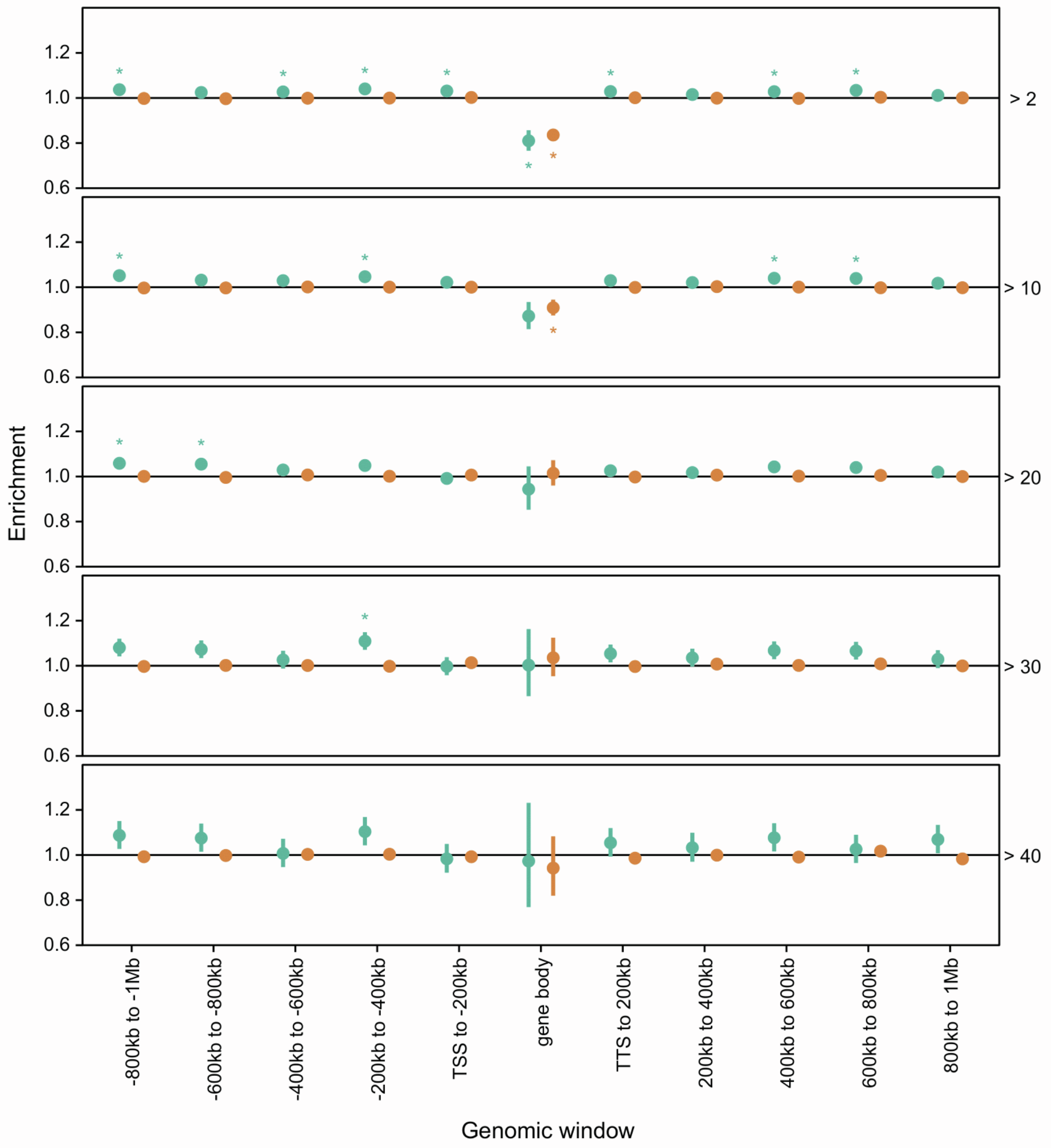

Figure S10. Enrichment of rare SNVs and indels across genomic windows and misexpression Z score thresholds.

Enrichment of rare (MAF < 1%) SNVs (orange) and indels (green) within 200 kb genomic windows and the body of the misexpressed gene across different misexpression Z score thresholds. Enrichments were calculated as the relative risk of having a nearby rare variant type given the misexpression status. The line at enrichment = 1 indicates no enrichment; Asterisks positioned either side of the line indicate significant enrichment or underenrichment after Bonferroni correction. Bars represent 95% Wald confidence intervals of the relative risk estimates.

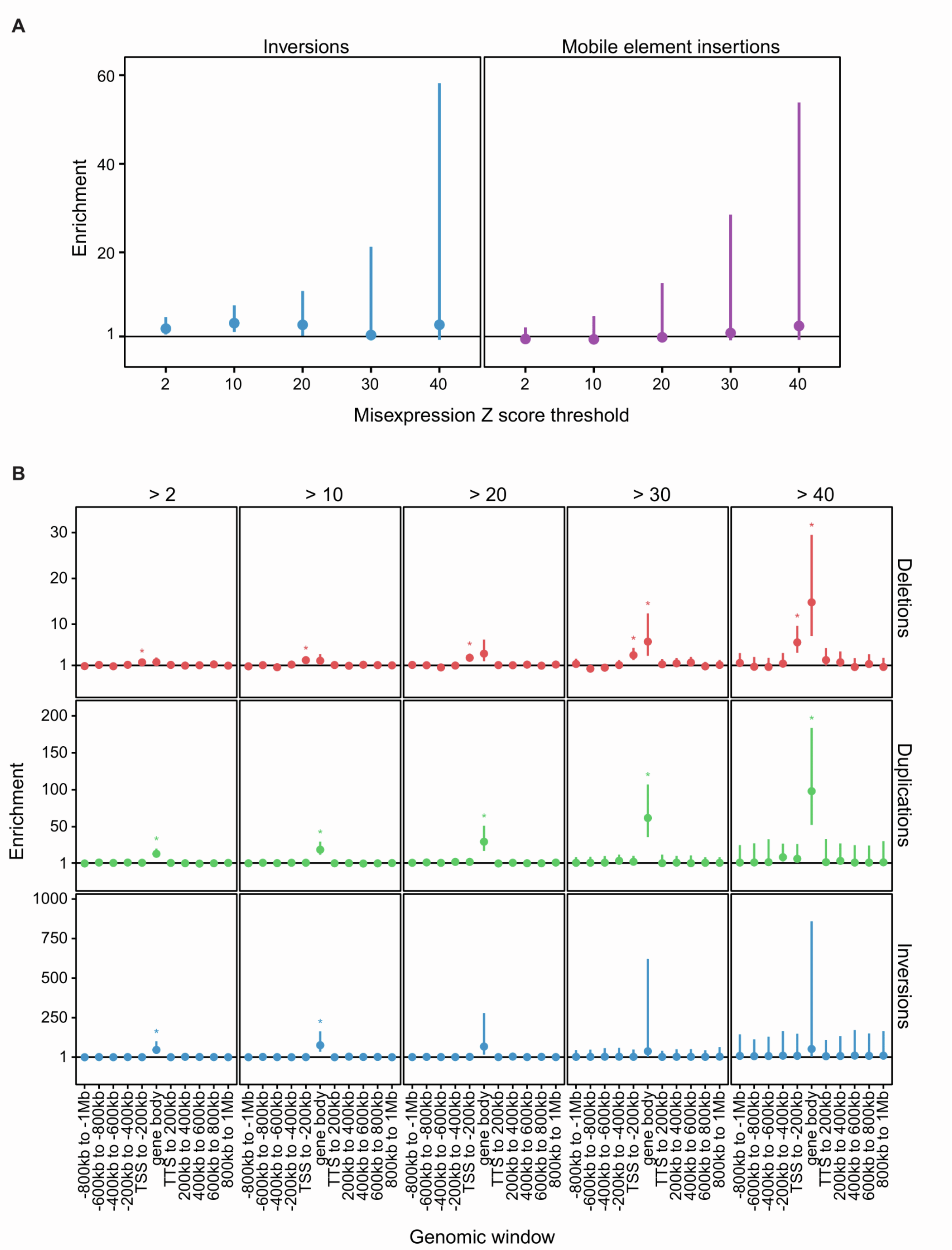

Figure S11. Enrichment of rare SV classes.

Enrichments were calculated as the relative risk of having a nearby variant type or consequence given the misexpression status. Bars represent 95% Wald confidence intervals of the relative risk estimates. The line at enrichment = 1 indicates no enrichment; stars positioned either side of the line indicate significant enrichment or underenrichment after Bonferroni correction. **A.)** Enrichment of rare (MAF < 1%) inversions and mobile element insertions in a ±200 kb window around the tested genes across different misexpression Z score thresholds. **B.)** Enrichment of rare (MAF < 1%) deletions, duplications,

and inversions within 200 kb genomic windows and the body of the misexpressed gene across different misexpression Z score thresholds.

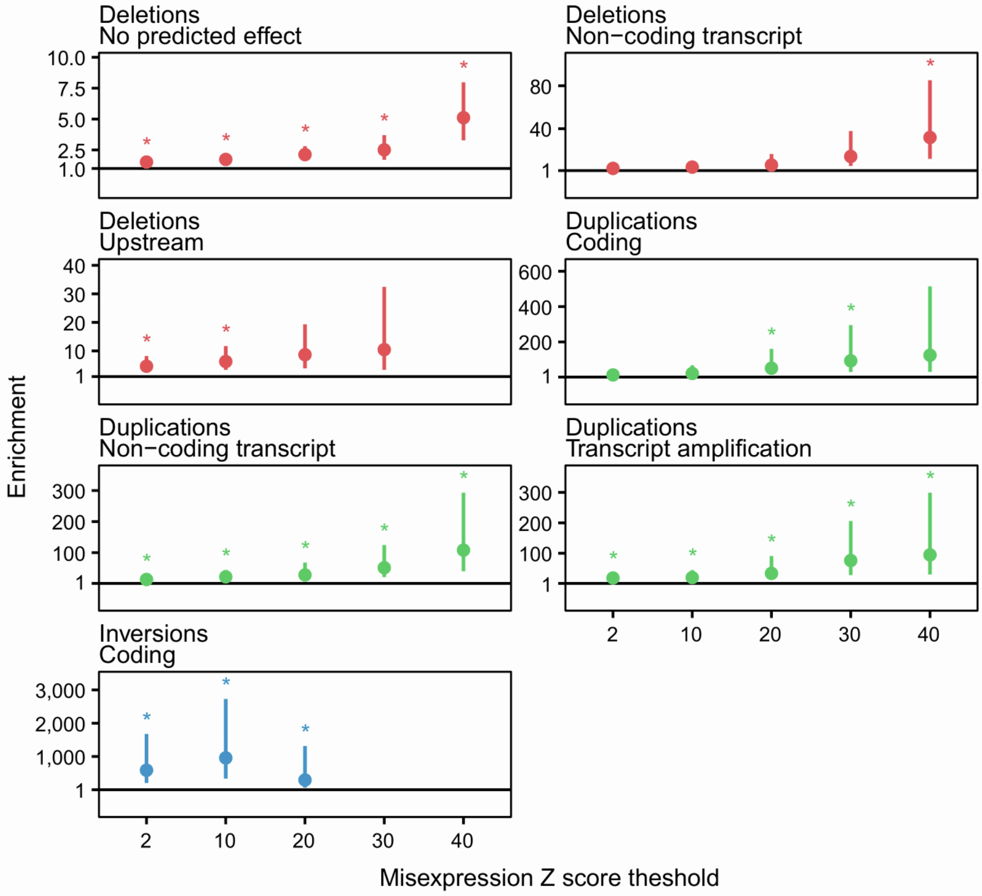

Figure S12. Enrichment of rare SVs stratified by their class and predicted VEP consequences across misexpression Z score thresholds.

Enrichments were calculated as the relative risk of having a nearby variant consequence given the misexpression status. The line at enrichment = 1 indicates no enrichment; asterisks positioned either side of the line indicate significant enrichment or underenrichment after Bonferroni correction. Bars represent 95% Wald confidence intervals of the relative risk estimates. Only SV consequences with at least one Bonferroni significant enrichment at any Z score threshold are shown. Missing points indicate tests failing to pass the nominal p-value threshold ($p \geq 0.05$).

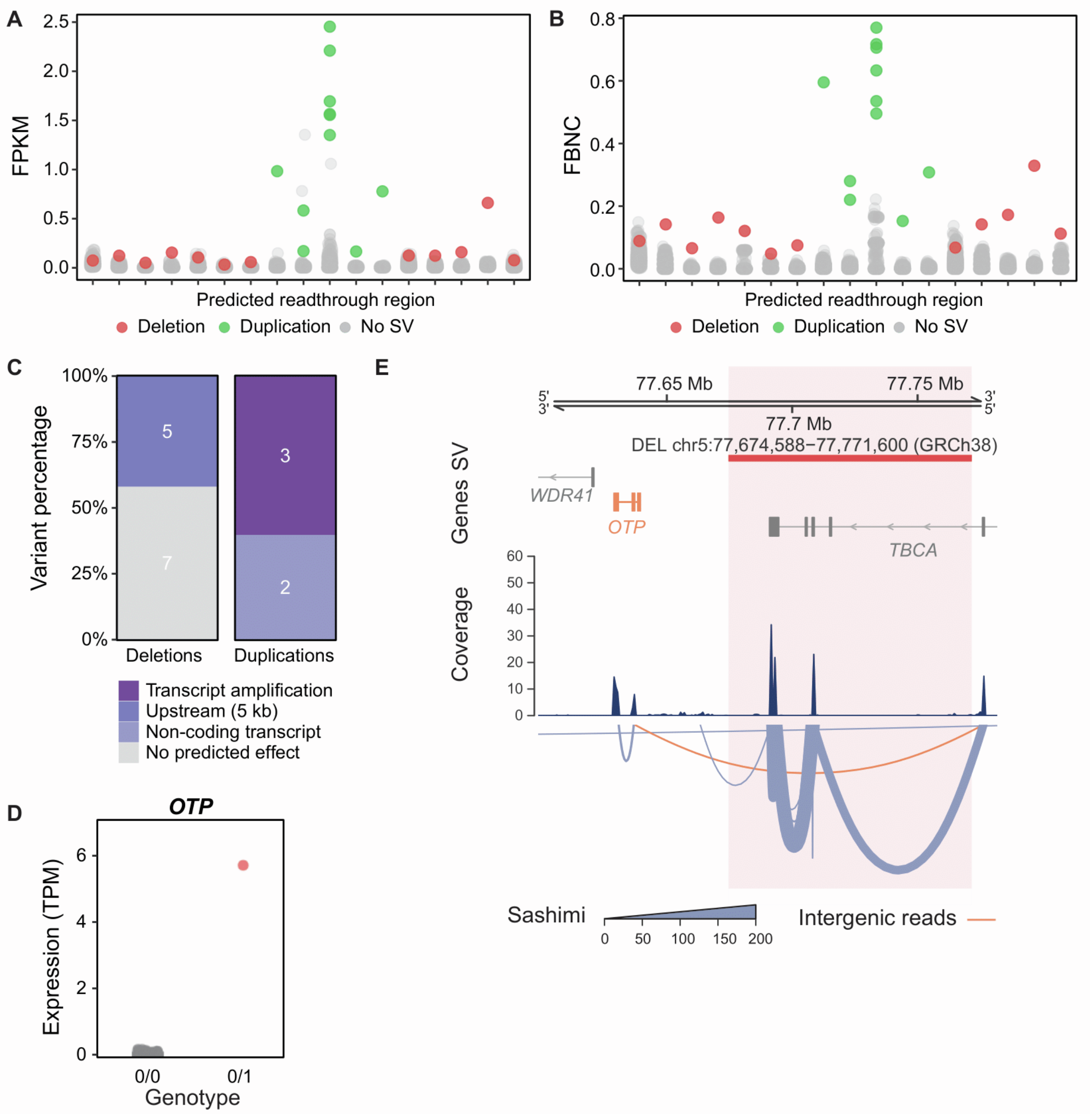

Figure S13. Transcriptional readthrough region FPKM and FBNC, transcription readthrough SV consequences and OTP misexpression.

A.) FPKM and **B.)** FBNC at the 17 predicted readthrough regions for samples with candidate transcriptional readthrough deletions (red) and duplications (green), as well as samples with no candidate SVs (gray). **C.)** Proportion of candidate transcriptional readthrough deletions and duplications by their predicted VEP consequence on the misexpressed gene**. D.)** Expression of *OTP* comparing samples with DEL chr5:77674588−77771600 (GRCh38) to samples without the deletion. Red color indicates samples passing the misexpression threshold TPM > 0.5 and Z score > 2 while gray samples are below this threshold. **E.)** Deletion of the 3' end of *TBCA* results in transcriptional readthrough. Transcriptional readthrough leads to *OTP* misexpression (orange gene) and intergenic splicing between *TBCA* and *OTP* (intergenic reads, orange). In the sashimi plot, the line width corresponds to the number of reads spanning a given junction.

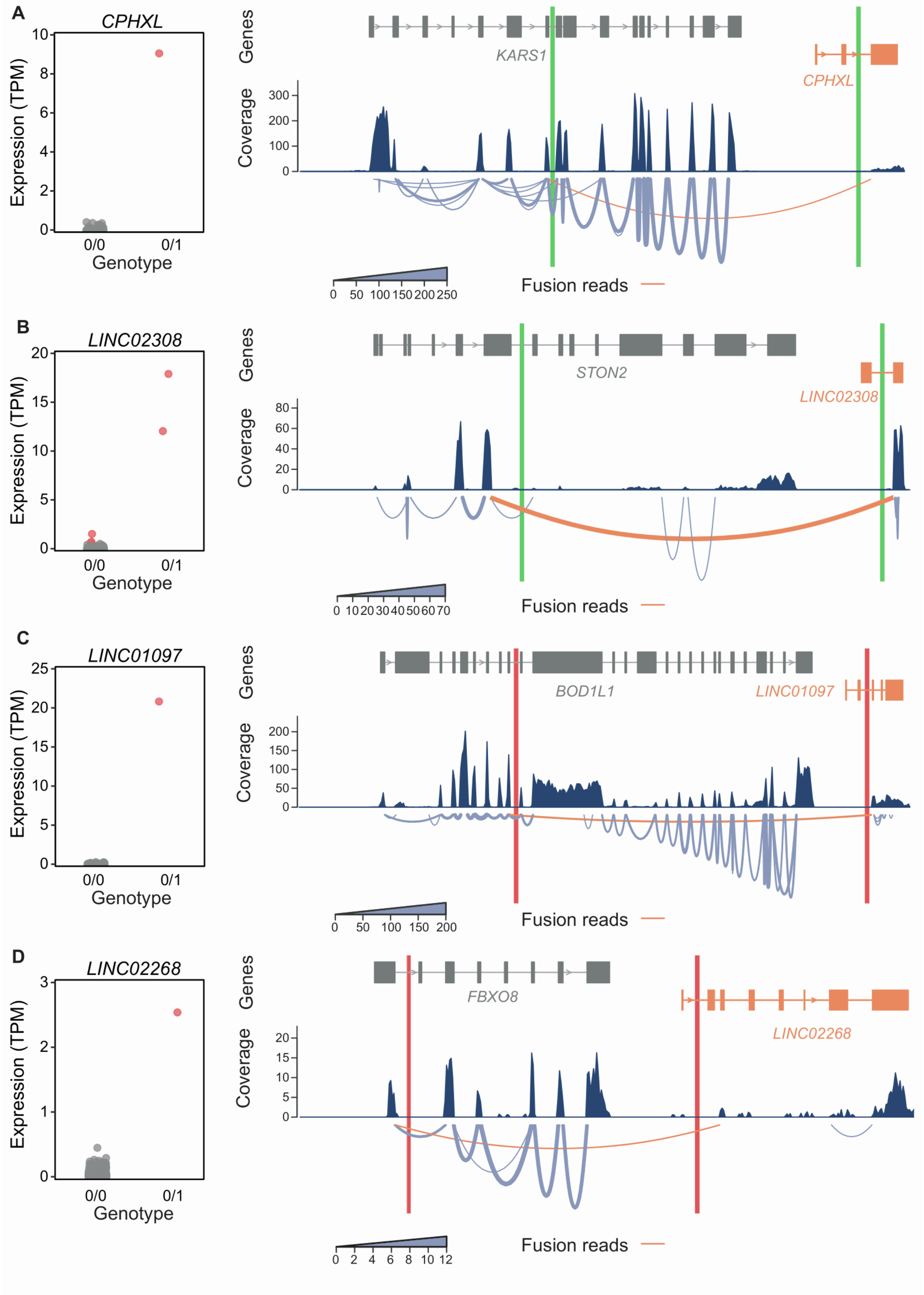

Figure S14. Examples of chimeric misexpression via transcript fusion.
Comparison of expression in samples with and without an SV associated with chimeric misexpression via transcript fusion alongside FusionInspector visualization of the fusion transcript in a sample with a misexpression-associated SV for **A.)** *CPHXL* misexpression via *KARS1–CPHXL* fusion in a sample with

DUP chr16:75636427-75717471 (GRCh38), **B.)** *LINC02308* misexpression via *STON2–LINC02308* fusion in samples with DUP chr14:81376672-81444942 (GRCh38), **C.)** *LINC01097* misexpression via *BOD1L1–LINC01097* fusion in a sample with DEL chr4:13529219-13608506 (GRCh38), and **D.)** *LINC02268* misexpression via *FBXO8–LINC02268* fusion in a sample with DEL chr4:174159287-174273823, (GRCh38). Red color indicates samples passing the misexpression threshold TPM > 0.5 and Z score > 2 while gray samples are below this threshold. In the sashimi plot, the line width corresponds to the number of reads spanning a given junction. The misexpressed gene and fusion reads are colored in orange. Deletion and duplication breakpoints are colored in red and green, respectively. Introns have been shortened for visualization and breakpoint positions have been approximated accordingly.

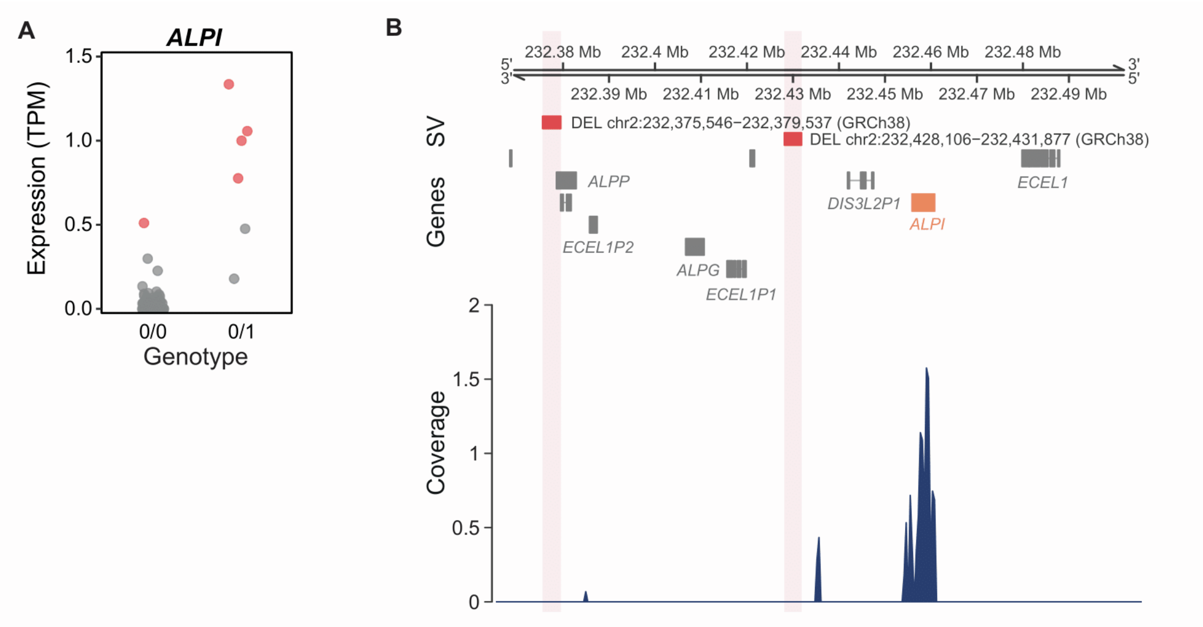

Figure S15. Intestinal alkaline phosphatase (ALPI) misexpression.

A.) Expression of *ALPI* in samples with DEL chr2:232375546-232379537 (GRCh38) and DEL chr2:232428106-232431877 (GRCh38) compared to samples without these deletions. Red color indicates samples passing the misexpression threshold TPM > 0.5 and Z score > 2 while gray samples are below this threshold. **B.)** Position of DEL chr2:232375546-232379537 (GRCh38) and DEL chr2:232428106-232431877 (GRCh38) relative to the misexpressed gene *ALPI* (orange gene). Deletions are marked in red.

Acknowledgements

Participants in the INTERVAL randomized controlled trial were recruited with the active collaboration of NHS Blood and Transplant England (https://www.nhsbt.nhs.uk/), which has supported field work and other elements of the trial. DNA extraction and genotyping were co-funded by the National Institute for Health and Care Research (NIHR), the NIHR BioResource (https://bioresource.nihr.ac.uk/) and the NIHR Cambridge Biomedical Research Centre (BRC-1215-20014). RNA-seq was funded as part of an alliance between the University of Cambridge and the AstraZeneca Centre for Genomics Research, and by the NIHR Cambridge Biomedical Research Centre (BRC-1215-20014). The academic coordinating center for INTERVAL was supported by core funding from the NIHR Blood and Transplant Research Unit (BTRU) in Donor Health and Genomics (NIHR BTRU-2014-10024); NIHR BTRU in Donor Health and Behaviour (NIHR203337); UK Medical Research Council (MR/L003120/1); British Heart Foundation (SP/09/002; RG/13/13/30194; RG/18/13/33946); and NIHR Cambridge BRC (BRC-1215-20014; NIHR203312). A complete list of the investigators and contributors to the INTERVAL trial is provided in Di Angelantonio et al.[1]. The academic coordinating center would like to thank blood donor center staff and blood donors for participating in the INTERVAL trial. This work was supported by Health Data Research UK, which is funded by the UK Medical Research Council, Engineering and Physical Sciences Research Council, Economic and Social Research Council, Department of Health and Social Care (England), Chief Scientist Office of the Scottish Government Health and Social Care Directorates, Health and Social Care Research and Development Division (Welsh Government), Public Health Agency (Northern Ireland), British Heart Foundation and Wellcome. The views expressed are those of the authors and not necessarily those of the NIHR or the Department of Health and Social Care.

Personal funding/acknowledgements:
T.V. was supported by a BBSRC iCASE Studentship partly funded by AstraZeneca (BB/V509425/1). A.T. was supported by the Wellcome Trust (PhD studentship 222548/Z/21/Z). E.P. was funded by the EU/EFPIA Innovative Medicines Initiative Joint Undertaking BigData@Heart grant 116074 and is funded by the NIHR BTRU in Donor Health and Behaviour (NIHR203337). J.D. holds a British Heart Foundation Professorship and a NIHR Senior Investigator Award. M.I. is supported by the Munz Chair of Cardiovascular Prediction and Prevention and the NIHR Cambridge Biomedical Research Centre (BRC-1215-20014; NIHR203312). M.I. was also supported by the UK Economic and Social Research Council (ES/T013192/1). A.S.B. has received grants outside of this work from AstraZeneca, Bayer, Biogen, BioMarin and Sanofi.

References

1. Di Angelantonio, E., Thompson, S.G., Kaptoge, S., Moore, C., Walker, M., Armitage, J., Ouwehand, W.H., Roberts, D.J., Danesh, J., and INTERVAL Trial Group (2017). Efficiency and safety of varying the frequency of whole blood donation (INTERVAL): a randomised trial of 45 000 donors. Lancet *390*, 2360–2371.
